# Functional mutation allele mining of plant architecture and yield-related agronomic traits and characterization of their effects in wheat

**DOI:** 10.1186/s12863-019-0804-2

**Published:** 2019-12-30

**Authors:** Huijun Guo, Hongchun Xiong, Yongdun Xie, Linshu Zhao, Jiayu Gu, Shirong Zhao, Yuping Ding, Luxiang Liu

**Affiliations:** Institute of Crop Sciences, Chinese Academy of Agricultural Sciences, National Engineering Laboratory of Crop Molecular Breeding, National Center of Space Mutagenesis for Crop Improvement, Beijing, China

**Keywords:** Wheat, Mutant resource, Mutation allele, Favorable allele, Plant architecture, Yield-related traits

## Abstract

**Background:**

Wheat mutant resources with phenotypic variation have been developed in recent years. These mutants might carry favorable mutation alleles, which have the potential to be utilized in the breeding process. Plant architecture and yield-related features are important agronomic traits for wheat breeders and mining favorable alleles of these traits will improve wheat characteristics.

**Results:**

Here we used 190 wheat phenotypic mutants as material and by analyzing their SNP variation and phenotypic data, mutation alleles for plant architecture and yield-related traits were identified, and the genetic effects of these alleles were evaluated. In total, 32 mutation alleles, including three pleiotropic alleles, significantly associated with agronomic traits were identified from the 190 wheat mutant lines. The SNPs were distributed on 12 chromosomes and were associated with plant height (PH), tiller number, flag leaf angle (FLA), thousand grain weight (TGW), and other yield-related traits. Further phenotypic analysis of multiple lines carrying the same mutant allele was performed to determine the effect of the allele on the traits of interest. PH-associated SNPs on chromosomes 2BL, 3BS, 3DL, and 5DL might show additive effects, reducing PH by 10.0 cm to 31.3 cm compared with wild type, which means that these alleles may be favorable for wheat improvement. Only unfavorable mutation alleles that reduced TGW and tiller number were identified. A region on chromosome 5DL with mutation alleles for PH and TGW contained several long ncRNAs, and their sequences shared more than 90% identity with cytokinin oxidase/dehydrogenase genes. Some of the mutation alleles we mined were colocalized with previously reported QTLs or genes while others were novel; these novel alleles could also result in phenotypic variation.

**Conclusion:**

Our results demonstrate that favorable mutation alleles are present in mutant resources, and the region between 409.5 to 419.8 Mb on chromosome 5DL affects wheat plant height and thousand grain weight.

## Background

In the past few decades, mutation induction has been used to improve crop varieties. Using this procedure, more than 3200 new mutant plant varieties have been bred from over 200 species worldwide (https://mvd.iaea.org/). The development of genomics and biological techniques has facilitated the use of mutation induction to elucidate the nature of mutations and for mining novel alleles and genes affecting target traits. Chemical mutagens can induce a high rate of single nucleotide polymorphism (SNP) variation, up to more than 5000 mutations on average in each M_2_ line of hexaploid wheat [1], while physical mutagens like fast neutrons and heavy ion beams induce more substitutions than small insertion-deletions or large deletions [2, 3]. Using an M_2_ population and reverse-genetic methodologies, such as TILLING (Targeting Induced Local Lesions in Genomes), novel alleles of known genes have been identified and functionally characterized in several plant species including wheat [4–8].

Wheat mutant resources have also been developed in the past decades from several different wild types [4, 9–12]. Various mutations affecting plant architecture, spike morphology, and yield-related traits have been identified, and there are many novel mutant genes/alleles available. In order to mine the mutated genes in these mutant lines, bi-parental genetic segregation populations, such as recombinant inbred lines (RILs) and near isogenic lines (NILs), need to be generated using a mutant as one of the parents. The process of developing genetic populations is very time-consuming, requiring at least two more years after the mutant is identified, and thus the speed with which mutants can be used is limited. A new methodology that could identify mutated genes/QTLs directly from the mutants should be developed which would be helpful in accelerating the gene mining process.

Plant architecture and yield-related traits are the main targets in wheat breeding and many genes/QTLs have been mapped that have dominant or/and additive effects. Over 20 wheat dwarfing genes (*Rht*) [13] are available, and more than half were produced by mutation induction. Among these *Rht* genes, *Rht1*, *Rht2*, *Rht8*, and *Rht9* have been fully utilized in modern cultivars [14, 15], and different mutant alleles of *Rht1* and *Rht2* resulted in variance in plant height [16, 17], thus enriching genetic diversity. Moreover, there were many other potential loci distributed on multiple chromosomes that regulated plant height [18]. Yield and yield-related traits like thousand grain weight (TGW) and grain number per spike (GNS) are thought to be quantitative, and their genetic loci are distributed on chromosomes throughout the whole genome [19]. However, because of allelic selection during evolution and breeding processes, genetic diversity has remarkably declined [20, 21]. All of these genes/loci could be mutated to produce novel alleles, and the discovery and mining of favorable alleles to improve genetic diversity would greatly benefit wheat breeding.

Genome-wide association studies (GWAS) have played a crucial role in gene discovery in landraces, cultivars, and elite line resources of rice [22], maize [23], sorghum [24], and wheat [25]. Combined with high-throughput microarrays, such as 15 K [26], 90 K [27, 28], 660 K (http://wheat.pw.usda.gov/ggpages/topics/Wheat660_SNP_array_developed_by_CAAS.pdf) and 820 K [29] SNP and diversity array technology (DArT) arrays, novel alleles and QTLs for important agronomic traits, including plant height (PH), yield-related traits, and spike and flag leaf architecture, have been identified in wheat [25, 30–32]. In addition, some of the favorable alleles for TGW and GNS have been widely utilized in modern breeding [33].

In the current study, we detected mutated alleles affecting plant architecture and yield-related traits in a wheat mutant resource using the GWAS method combined with *t*-testing, and the genetic effects of mutated alleles were further evaluated. These results will improve mutant allele identification and the mining of novel mutation alleles affecting agronomic traits. The identification of favorable mutated alleles will in turn aid efforts to increase genetic diversity and improve wheat.

## Results

### Correlation between yield- and plant architecture-related traits

Correlation analysis (Table 1) showed that PH was significantly and positively correlated with the other four plant architecture-related traits, including pre-winter, maximum, and effective tiller numbers (PWT, MT, and ET, respectively), and flag leaf angle (FLA); ET was more highly correlated with MT than with PWT. TGW was significantly and positively correlated with all traits except FLA and spikelet density (SD), but the correlation coefficients were not very high.

**Table 1:** The correlation coefficients for plant architecture- and yield-related traits of wheat mutant lines averaged across environments

Trait	MT	ET	PWT	FLA	PH	SL	GNS	NSL	SD
ET	0.9096***								
PWT	0.7967***	0.7633***							
FLA	−0.1409	−0.1429*	−0.0329						
PH	0.3553***	0.3807***	0.4990***	0.3313***					
SL	−0.0724	−0.1085	0.1665*	0.3571***	0.5096***				
GNS	−0.1425*	− 0.1790*	−0.0493	0.0843	0.0718	0.2804***			
NSL	0.0595	0.018	0.2284**	0.1461*	0.2607***	0.4350***	0.5834***		
SD	0.1646*	0.1686*	0.0022	−0.3500***	−0.4126***	−0.8178***	0.0071	0.0784	
TGW	0.1479*	0.1614*	0.3179***	−0.0109	0.4755***	0.1793*	0.1439*	0.2030**	−0.0727

*MT* maximum tiller numbers, *ET* effective tiller numbers, *PWT* pre-winter tiller numbers, *FLA* flag leaf angle, *PH* plant height, *SL* spike length, *GNS* grain numbers per spike, *NSL* spikelet number per spike, *SD* spikelet density, *TGW* thousand grain weight

*significant at *P* < 0.05 level; ** significant at *P* < 0.01 level; *** significant at *P* < 0.001 level

### SNPs potentially associated with plant architecture- and yield-related traits under different environments

The principal component analysis (PCA) indicated that the population was divided into five subpopulations (Additional file 1: Figure S1). High linkage disequilibrium (LD) was observed in the mutant population, with an average r^2^ of 0.91 at *p* ≤ 0.001.

A total of 150 potential significant SNPs were detected at the given *P* value threshold (0.001) in at least one environment by genome-wide association analysis (Additional file 2: Table S1; Additional file 3: Figure S2; Additional file 4: Figure S3). The SNPs were distributed on all chromosomes except chr. 1D, 2D, and 4D, with 35 located on chr. 3B. Among these significant SNPs, 62 were potentially associated with plant architecture-related traits, including eight SNPs associated with PH, five with PWT, 15 with MT, 27 with ET, and seven with FLA; 88 were potentially associated with yield-related traits, including 23 associated with TGW, 21 with spike length (SL), 13 with GNS, 15 with SD, and 16 with spikelet number per spike (NSL).

### Candidate SNPs related to plant architecture- and yield-related traits

**Fig. 1:**
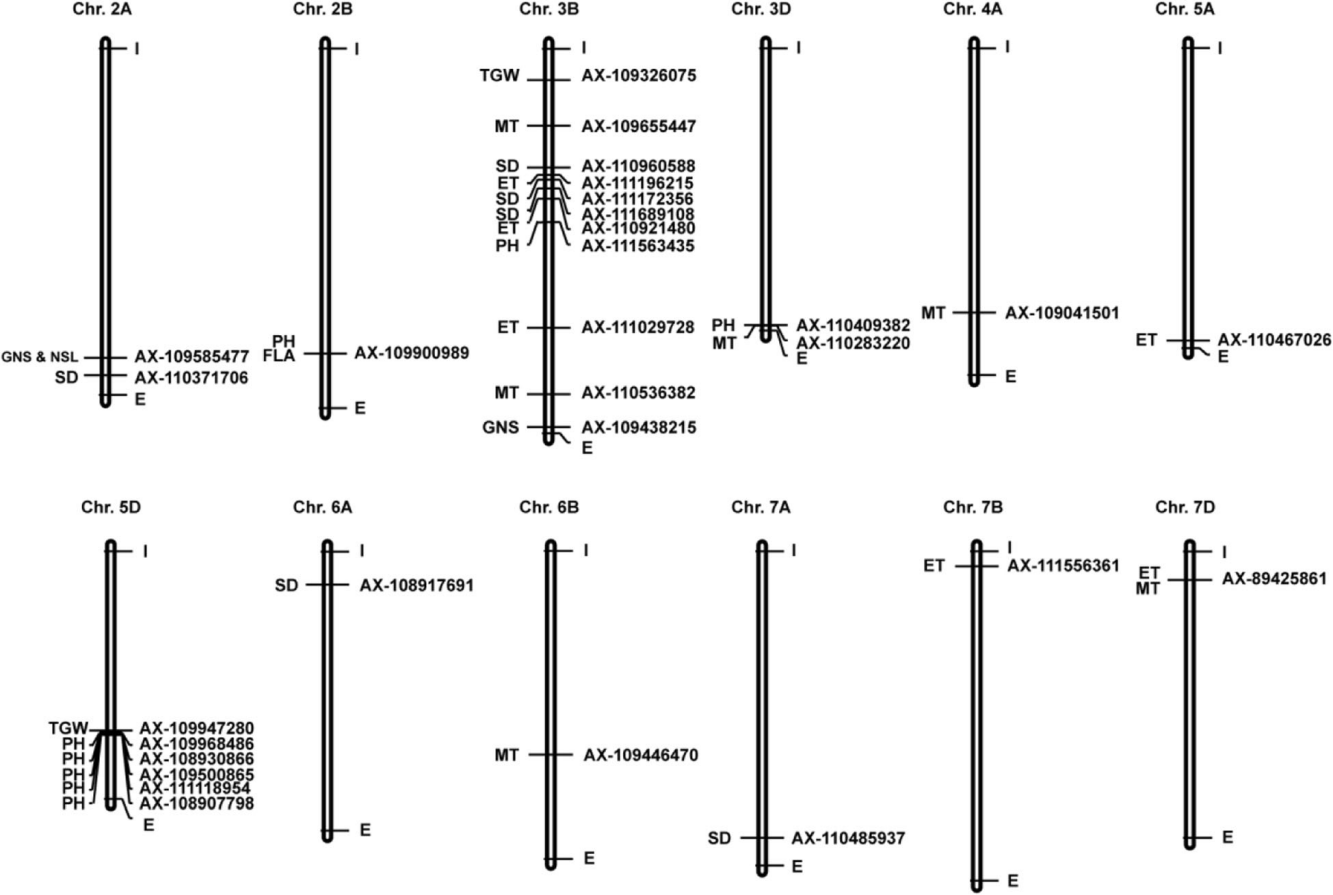
Relative physical position of significantly associated SNPs on chromosomes. The long black rectangles represent the chromosomes. Significantly associated SNPs are shown on the right side of the chromosome. The traits associated with the SNP are shown on the left side of the chromosome. I: the beginning of the chromosome; E: the end of chromosome

Based on *t*-tests, there was a significant difference in the phenotypes of plants with the wild type (WT) and mutation alleles in more than 50% of the environments for 32 out of the 150 SNPs (21.34%) (Table 2), and these SNPs were distributed in clusters on 12 chromosomes (Fig. 1). Among the plant architecture-related traits, all eight SNPs distributed on chr. 2B, 3B, 3D, and 5D were significantly associated with PH, and the percent variance explained (PVE) ranged from 7.68–9.40%. Six out of 15 SNPs (40.00%) distributed on chr. 3B, 3D, 4A, 6B, and 7D were significantly associated with MT, and PVE ranged from 6.19 to 10.23%. Six out of 27 SNPs (22.23%) distributed on chr. 3B, 5A, 7B, and 7D were significantly associated with ET, and PVE ranged from 8.21 to 11.39%. One out of seven SNPs (14.29%) on chr. 2B was significantly associated with FLA, and the PVE was 6.18%.

**Table 2:** Candidate SNPs that were significantly associated with a trait in more than 50% of environments based on *t*-tests

Trait	Marker	Chr	Pos	*P*	PVE	*p* value of *t*-test					
						2015C	2015H	2016C	2016H	2017H	Ac-Env
PH	AX-109900989	2B	679,577,716	6.80E-05	0.08639	4E-06	2E-06	4E-06	1E-05	4E-05	1E-06
PH	AX-111563435	3B	374,142,031	7.47E-04	0.07895	0.45	0.026	0.002	0.003	4E-04	0.005
PH	AX-110409382	3D	603,468,706	6.77E-04	0.07683	0.127	1E-03	3E-05	7E-04	3E-06	2E-04
PH	AX-109968486	5D	414,844,244	2.66E-04	0.09143	0.002	3E-04	6E-07	1E-04	2E-07	7E-06
PH	AX-108930866	5D	417,084,071	6.87E-04	0.08045	0.002	3E-04	1E-06	2E-04	3E-07	1E-05
PH	AX-109500865	5D	418,685,224	2.66E-04	0.09143	0.002	3E-04	6E-07	1E-04	2E-07	7E-06
PH	AX-111118954	5D	419,371,841	6.98E-04	0.08027	0.002	4E-04	1E-06	2E-04	3E-07	1E-05
PH	AX-108907798	5D	419,809,508	2.14E-04	0.09397	6E-04	4E-05	7E-08	2E-05	5E-08	1E-06
MT	AX-109655447	3B	165,293,369	3.69E-04	0.09462	0.935	0.323	0.035	0.021	8E-04	0.018
MT	AX-110536382	3B	746,271,373	5.54E-04	0.0839	0.146	0.031	0.002	0.002	6E-04	0.002
MT	AX-110283220	3D	604,711,079	7.01E-04	0.08781	0.808	0.419	0.018	0.002	5E-05	0.006
MT	AX-109041501	4A	601,871,802	8.21E-04	0.06193	0.501	0.053	0.023	0.022	9E-05	0.005
MT	AX-109446470	6B	476,960,396	1.13E-04	0.10228	0.835	0.261	0.023	0.002	0.036	0.067
MT	AX-89425861	7D	63,824,313	4.87E-04	0.09204	5E-05	1E-05	0.002	2E-04	9E-05	3E-05
ET	AX-111196215	3B	271,864,887	4.56E-06	0.11394	0.099	0.089	0.049	0.006	0.012	0.004
ET	AX-110921480	3B	323,802,217	4.99E-04	0.08531	0.052	0.049	0.017	0.091	0.004	0.002
ET	AX-111029728	3B	602,229,430	8.00E-04	0.08208	0.034	0.063	0.012	0.064	0.002	0.001
ET	AX-110467026	5A	692,057,838	5.37E-04	0.08326	0.016	0.196	0.018	0.22	0.035	0.007
ET	AX-111556361	7B	34,297,311	4.41E-04	0.08314	0.014	0.097	0.014	0.028	0.082	0.003
ET	AX-89425861	7D	63,824,313	2.41E-04	0.09777	4E-05	9E-07	3E-04	0.002	1E-04	7E-06
FLA	AX-109900989	2B	679,577,716	7.82E-04	0.06175	0.009	0.007	4E-07	1E-06		1E-06
TGW	AX-109326075	3B	67,251,955	3.22E-05	0.11594	0.018	7E-04	3E-04	0.013	0.125	7E-04
TGW	AX-109947280	5D	409,567,559	5.46E-04	0.08225	0.002	0.021	0.001	0.013	0.045	1E-03
GNS	AX-109585477	2A	695,514,686	1.15E-04	0.10446			2E-05	0.045		4E-04
GNS	AX-109438215	3B	817,259,816	3.57E-06	0.14869			0.002	0.031		0.003
NSL	AX-109585477	2A	695,514,686	8.74E-04	0.07398	0.723	0.063	0.009	0.004		0.006
SD	AX-110371706	2A	734,684,363	5.07E-04	0.07958	9E-07	1E-05	5E-07	2E-06		5E-09
SD	AX-110960588	3B	255,902,481	5.50E-04	0.07765	0.665	0.154	0.007	0.021		0.03
SD	AX-111172356	3B	282,202,013	5.40E-04	0.07766	0.585	0.098	0.005	0.014		0.02
SD	AX-111689108	3B	301,074,069	5.37E-04	0.07774	0.572	0.099	0.01	0.024		0.03
SD	AX-108917691	6A	73,473,707	5.90E-04	0.07935	0.811	0.014	0.005	0.192		0.061
SD	AX-110485937	7A	671,726,465	5.30E-04	0.07788	0.613	0.019	0.017	0.099		0.039

PVE (%): phenotypic variation explained by the SNP

*PH* plant height, *MT* maximum tiller numbers, *ET* effective tiller numbers, *FLA* flag leaf angle, *TGW* thousand grain weight, *GNS* grain numbers per spike, *NSL* spikelet number per spike, *SD* spikelet density

For the yield-related traits, two out of 23 SNPs (8.70%) distributed on chr. 3B and 5D were significantly associated with TGW based on *t*-tests, and PVE ranged from 8.23 to 11.59%. Two out of 13 SNPs (15.39%) distributed on chr. 2A and 3B were significantly associated with GNS, and PVE ranged from 10.45 to 14.87%. One out of 16 SNPs (6.25%) on chr. 2A was significantly associated with NSL and explained 7.40% of the phenotypic variation. Six out of 15 SNPs (40.00%) distributed on chr. 2A, 3B, 6A, and 7A were significantly associated with SD, and PVE ranged from 7.77 to 7.96%.

There were three candidate SNPs showing pleiotropic effects. SNP AX-109900989 was significantly associated with both PH and FLA, AX-89425861 was associated with both MT and ET, and AX-109655447 was associated with GNS and NSL.

### Effects of candidate mutation alleles on plant architecture and yield-related traits

#### Plant height

Among the 190 mutant lines, 158 lines carried only the mutant allele of SNP AX-109900989; five lines carried the mutant alleles of both SNP AX-110409382 and SNP AX-111563435, which are located on chr. 3B and 3D, respectively; and 11 lines carried the mutant alleles of all eight significant SNPs located on chr. 2B, 3B, 3D, and 5D (Additional file 5: Table S2 and Table 3). The physical distance between the five SNPs on chr. 5D is about 4.97 Mb (Table 3).

**Table 3:** *P* values for *t*-tests between WT and mutants carrying candidate SNPs for plant height

SNP	Chromosome	Position	Mutation allele	Line number	Env	*P* value	Average	Sta Dev	Min	Max
AX-109900989	2B	679,577,716	GG	158	2015C	0.182242	54.8	9.71	24.9	90.7
					2015H	0.064806	67.5	10.98	33.0	96.0
					2016C	0.003819	77.8	11.51	41.0	107.8
					2016H	4.44E-06	73.4	11.93	33.7	103.3
					2017H	0.000261	80.6	11.54	47.8	116.0
					Ac-Env	0.000153	71.2	10.42	40.9	103.1
AX-111563435	3B	374,142,031	GG	5	2015C	0.236392	59.2	3.33	55.5	63.3
AX-110409382	3D	603,468,706	CC		2015H	0.056725	66.4	1.92	62.8	68.0
					2016C	0.001168	77.5	2.76	74.4	81.0
					2016H	0.000134	71.8	2.00	68.2	73.8
					2017H	0.004186	77.3	5.54	67.0	82.7
					Ac-Env	0.001217	70.7	1.41	68.2	72.6
AX-109900989	2B	679,577,716	GG	11	2015C	0.095321	49.0	5.95	38.9	58.2
AX-111563435	3B	374,142,031	GG		2015H	0.008369	56.8	6.03	46.8	66.5
AX-110409382	3D	603,468,706	CC		2016C	5.86E-07	62.5	8.01	47.0	81.2
AX-109968486	5D	414,844,244	TT		2016H	5.88E-06	61.4	9.41	45.4	81.5
AX-108930866	5D	417,084,071	TT		2017H	1.99E-07	64.1	7.34	53.8	80.8
AX-109500865	5D	418,685,224	TT		Ac-Env	1.28E-07	59.1	6.54	47.0	72.8
AX-111118954	5D	419,371,841	GG							
AX-108907798	5D	419,809,508	CC							

**Fig. 2:**
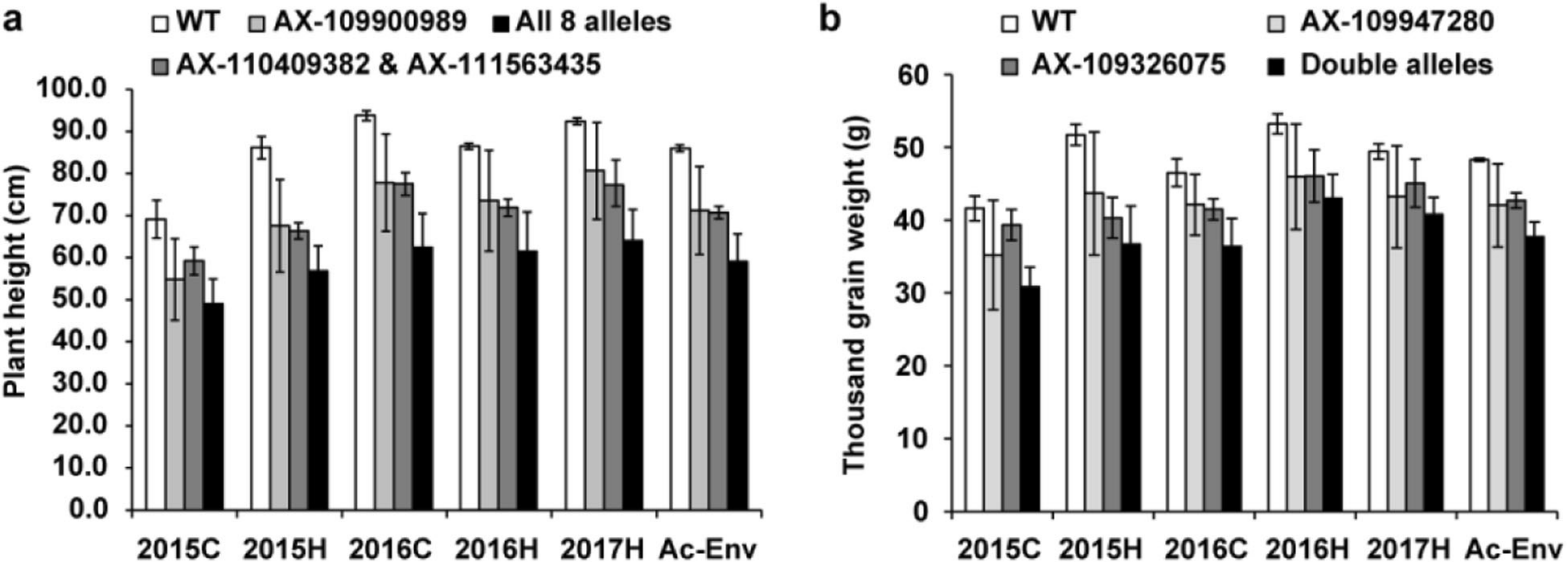
Average plant height and thousand grain weight of wild type (WT) and mutant lines carrying different mutation alleles. **a**, Plant height. All eight alleles indicate lines carrying the mutant alleles of AX-109900989, AX-111563435, AX-110409382, AX-109968486, AX-108930866, AX-109500865, AX-111118954, and AX-108907798. **b**, Thousand grain weight. Double alleles indicate lines carrying the mutant alleles of AX-109947280 and AX-109326075

The presence of single mutation alleles and pyramided mutation alleles resulted in a significant reduction of PH compared with WT (Fig. 2a). The average PH of the mutants only carrying the mutation allele of AX-109900989 was 11.8 cm to 18.6 cm lower than that of WT in different environments. PH of lines carrying the mutant alleles of both AX-110409382 and AX-111563435 was reduced by 10.0 cm to 19.8 cm, and the PH of lines carrying all eight mutation alleles was reduced by 20.2 cm to 31.3 cm, with the pyramided alleles implying additive effects.

#### Thousand grain weight

There were three and seven mutant lines carrying single mutation alleles of AX-109326075 (chr. 3B) and AX-109947280 (chr. 5D), respectively. The TGW of these plants were lower than that of WT, but these differences were not significant (Additional file 6: Table S3 and Table 4). Six mutant lines carried both mutation alleles, and the TGW of these lines was significantly lower than that of WT (by 3.48 to 16.84 g) in different environments (Additional file 6: Table S3, Table 4, and Fig. 2b). The effects of these two alleles were additive. The physical positions of both SNPs are located far away from the other SNPs on chr. 3B and 5D that affect PH.

**Table 4:** *P* values for *t*-tests between WT and mutants carrying candidate SNPs for thousand grain weight

SNP	Chromosome	Position	Mutation allele	Line number	Env	*P* value	Average	Sta Dev	Min	Max
AX-109326075	3B	67,251,955	GG	3	2015C	0.405847	39.35	2.11	37.4	42.3
					2015H	0.018723	40.33	2.78	36.5	42.8
					2016C	0.175195	41.47	1.45	39.6	43.1
					2016H	0.092346	46.05	3.58	41.0	49.1
					2017H	0.196281	45.05	3.29	40.5	48.3
					Ac-Env	0.0129	42.68	1.05	41.3	43.8
AX-109947280	5D	409,567,559	TT	7	2015C	0.1132035	35.22	7.51	21.4	42.5
					2015H	0.0687416	43.67	8.45	27.8	51.2
					2016C	0.1856102	42.12	4.16	34.7	47.7
					2016H	0.0605804	45.97	7.23	30.3	55.4
					2017H	0.0804989	43.20	7.01	32.3	55.2
					Ac-Env	0.035889	42.03	5.70	29.8	47.4
AX-109326075	3B	67,251,955	GG	6	2015C	0.029895	30.87	2.66	25.6	34.5
AX-109947280	5D	409,567,559	TT		2015H	0.0020815	36.72	5.22	29.5	43.4
					2016C	0.0299556	36.39	3.85	29.4	41.2
					2016H	0.0083509	43.01	3.27	37.5	48.4
					2017H	0.0085744	40.82	2.24	37.0	43.7
					Ac-Env	5.371E-05	37.76	2.01	34.6	40.4

#### Spikelet density

**Fig. 3:**
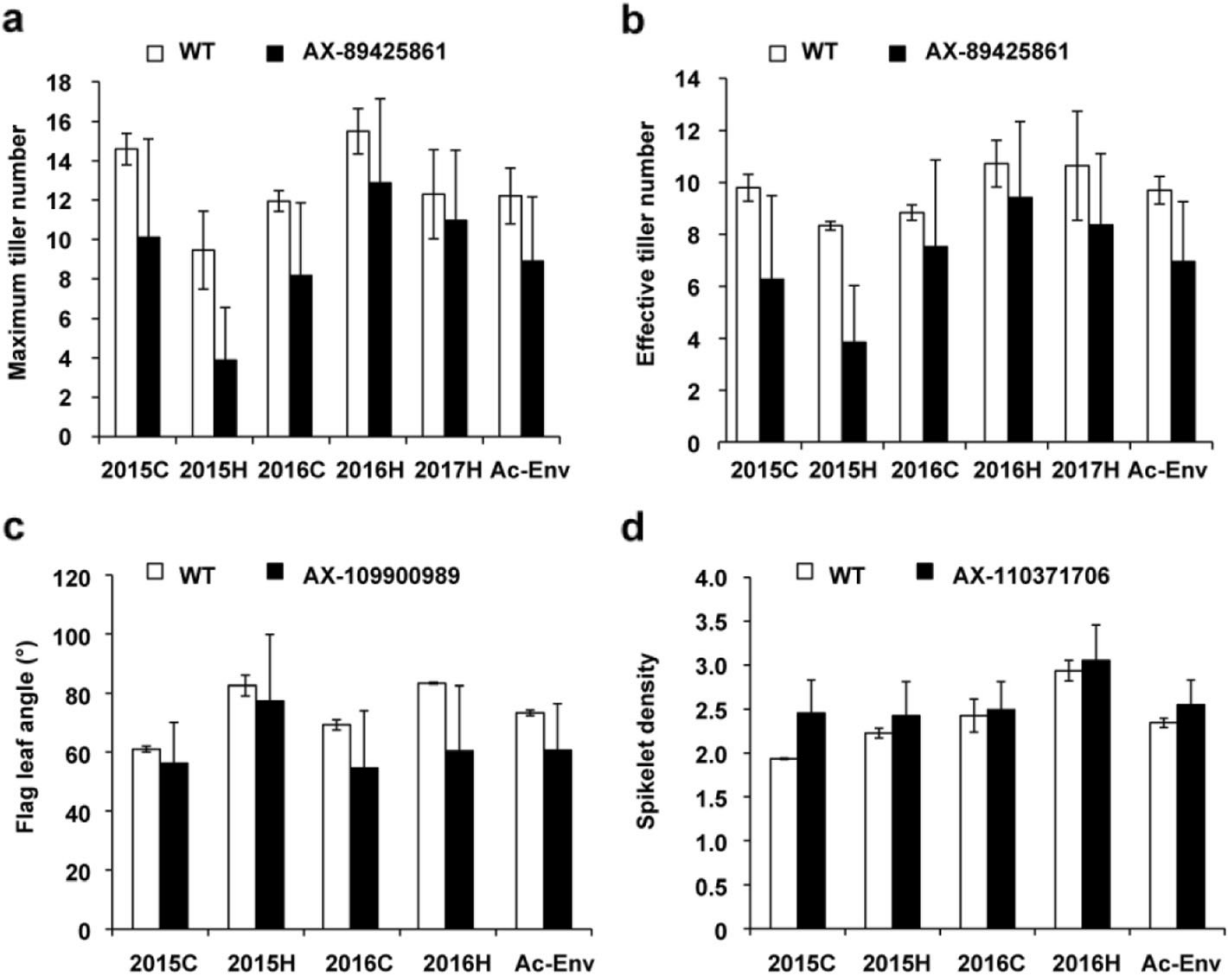
Average phenotypic values of wild type (WT) and mutant lines carrying different mutation alleles. **a**, Maximum tiller number; **b**, Effective tiller number; **c**, Flag leaf angle; **d**, Spikelet density

The average SD of the mutants carrying the mutation allele of AX-110371706 was higher than that of the WT (Table 5, Fig. 3d, and Additional file 10: Table S7), with the increase ranging from 0.07 to 0.52 in different environments.

**Table 5:** *P* values for *t*-tests between WT and mutants carrying candidate SNPs for maximum tiller number

Trait	SNP	Mutation allele	Line number	Env	*P* value	Average	Sta Dev	Min	Max
MT	AX-89425861	GG	67	2015C	0.030429	10.11	5.00	1.0	21.2
				2015H	0.209076	3.89	2.67	0.5	11.8
				2016C	0.012518	8.17	3.69	1.0	17.9
				2016H	0.218807	12.88	4.25	1.2	25.2
				2017H	0.661201	10.97	3.58	2.3	21.5
				Ac-Env	0.235913	8.89	3.28	1.4	19.1
ET	AX-89425861	GG	67	2015C	0.019426	6.27	3.22	0.1	14.9
				2015H	6.87E-09	3.85	2.17	0.6	10.0
				2016C	0.031796	7.52	3.35	0.7	15.8
				2016H	0.363565	9.43	2.93	0.1	15.9
				2017H	0.470211	8.36	2.76	1.6	15.0
				Ac-Env	0.062644	6.95	2.31	1.0	11.1
SD	AX-110371706	TT	66	2015C	2.33E-16	2.46	0.37	1.47	3.53
				2015H	0.077025	2.43	0.39	1.21	3.96
				2016C	0.78042	2.49	0.32	1.67	3.07
				2016H	0.488383	3.05	0.41	1.95	3.83
				Ac-Env	0.083838	2.55	0.28	1.58	3.04
FLA	AX-109900989	GG	157	2015C	0.025269	56.27	13.86	27.0	107.0
				2015H	0.350709	77.41	22.40	37.5	144.5
				2016C	0.006889	54.60	19.40	17.5	126.5
				2016H	6.93E-26	60.41	22.01	19.0	134.5
				Ac-Env	8.54E-05	60.69	15.69	22.3	120.3

*MT* maximum tiller numbers, *ET* effective tiller numbers, *SD* spikelet density, *FLA* flag leaf angle

#### Pleiotropic SNPs

There were 67 mutant lines that only carried the mutation allele of the candidate SNP AX-89425861, and the MT and ET of these lines, especially ET, were significantly lower than those of WT in some environments (Table 5, Fig. 3a, b, Additional file 7: Table S4 and Additional file 8: Table S5). The reduction in MT and ET ranged from 1.3 to 5.6 and 1.3 to 4.5, respectively.

AX-109900989 is another pleiotropic SNP. Compared with WT, lines carrying this mutation allele not only had lower PH, but also a lower FLA, which was reduced by 4.7 to 22.8° compared with WT (Table 5, Fig. 3c, Additional file 9: Table S6).

### Genes flanking the mutation alleles

There were no candidate SNPs located in genic sequence, so the flanking genes of each SNP were further searched (Table 6). The functions of these genes are related to imidazoleglycerol-phosphate dehydratase, protein kinase domain, and Myb-like DNA-binding domain, for example. Because of the very short physical distance of the five SNPs on chr. 5DL, the genes between SNPs AX-109968486 and AX-108907798, 42 in total, were further blasted (Table 7). Among them, gene TraesCS5D02G323500 was known as an Auxin-Indole-3-Acetic Acid (Aux-IAA) family transcription factor.

**Table 6:** The nearest genes to the candidate SNPs and their annotations

Trait	SNP	Gene	Annotation
PH	AX-109900989	TraesCS2B02G481000	Imidazoleglycerol-phosphate dehydratase
PH	AX-111563435	TraesCS3B02G238800	Protein kinase domain
PH	AX-110409382	TraesCS3D02G522200	PREDICTED: *Aegilops tauschii subsp. tauschii* uncharacterized LOC109756842 (LOC109756842), mRNA
PH	AX-109968486	TraesCS5D02G322900	Cytochrome P450
PH	AX-108930866	TraesCS5D02G325400	Domain of unknown function - DUF702
PH	AX-109500865	TraesCS5D02G326400	Domain of unknown function - DUF4220
PH	AX-111118954	TraesCS5D02G326900	*Triticum aestivum* cultivar Chinese Spring chloroplast, complete genome
PH	AX-108907798	TraesCS5D02G327000	PREDICTED: *Aegilops tauschii* subsp. *tauschii* uncharacterized LOC109776055 (LOC109776055), mRNA
MT	AX-109446470	TraesCS6B02G265200	Myb-like DNA-binding domain
MT	AX-89425861	TraesCS7D02G105200	–
ET	AX-111029728	TraesCS3B02G383800	Protein kinase domain
FLA	AX-109900989	TraesCS2B02G481000	Imidazoleglycerol-phosphate dehydratase
TGW	AX-109326075	TraesCS3B02G101900	R3H domain
TGW	AX-109947280	TraesCS5D02G315300	Ring finger domain
GNS	AX-109585477	TraesCS2A02G446500	RWD domain
GNS	AX-109438215	TraesCS3B02G595600	WD domain - G-beta repeat
NSL	AX-109585477	TraesCS2A02G446500	RWD domain
SD	AX-110371706	TraesCS2A02G507700	FAE1 -Type III polyketide synthase-like protein

**Table 7:** The genes between SNPs AX-109968486 and AX-108907798 on chr. 5DL and their annotations

SNPs	Gene	Annotation
AX-109968486		
	TraesCS5D02G323000	Transcription factor subunit Med10 of Mediator complex
	TraesCS5D02G323100	Homeobox domain
	TraesCS5D02G323200	PREDICTED: *Aegilops tauschii* subsp. *tauschii* 30S ribosomal protein S31, mitochondrial (LOC109733635), mRNA
	TraesCS5D02G323300	Kinesin motor domain
	TraesCS5D02G323400	B-box zinc finger
	TraesCS5D02G323500	AUX -IAA family
	TraesCS5D02G323600	U-box domain
	TraesCS5D02G323700	Glycosyl hydrolase family 9
	TraesCS5D02G323800	Cytochrome P450
	TraesCS5D02G323900	Cytochrome P450
	TraesCS5D02G324000	DOMON domain
	TraesCS5D02G324100	Leucine rich repeat
	TraesCS5D02G324200	WD domain - G-beta repeat; WD40 associated region in TFIID subunit
	TraesCS5D02G324300	Cytochrome P450
	TraesCS5D02G324400	Cytochrome P450
	TraesCS5D02G324500	SWIB -MDM2 domain, Plus-3 domain, GYF domain
	TraesCS5D02G324600	UDP-glucoronosyl and UDP-glucosyl transferase
	TraesCS5D02G324700	Glycosyl transferase family 2
	TraesCS5D02G324800	ThiF family
	TraesCS5D02G324900	PREDICTED: *Aegilops tauschii* subsp. *tauschii* uncharacterized LOC109778141
	TraesCS5D02G325000	Peptidase inhibitor I9
	TraesCS5D02G325100	Oxidoreductase-like protein - N-terminal
	TraesCS5D02G325200	RNA recognition motif 2
	TraesCS5D02G325300	PREDICTED: *Aegilops tauschii* subsp. *tauschii* protein MEI2-like 6
	TraesCS5D02G325400	Domain of unknown function - DUF702
AX-108930866		
	TraesCS5D02G325500	PREDICTED: *Aegilops tauschii* subsp. *tauschii* xylanase inhibitor protein 1-like
	TraesCS5D02G325600	Protein kinase domain
	TraesCS5D02G325700	Domain of unknown function - DUF2828
	TraesCS5D02G325800	PREDICTED: *Aegilops tauschii* subsp. *tauschii* polygalacturonate 4-alpha-galacturonosyltransferase-like
	TraesCS5D02G325900	PREDICTED: *Aegilops tauschii* subsp. *tauschii* protein STAY-GREEN, chloroplastic-like
	TraesCS5D02G326000	PREDICTED: *Aegilops tauschii* subsp. *tauschii* 60S ribosomal protein L36a (LOC109748907), mRNA
	TraesCS5D02G326100	Late embryogenesis abundant protein
	TraesCS5D02G326200	Response regulator receiver domain
	TraesCS5D02G326300	Pentatricopeptide repeat domain
	TraesCS5D02G326400	Domain of unknown function - DUF4220
AX-109500865		
	TraesCS5D02G326500	Alpha -beta hydrolase family
	TraesCS5D02G326600	Myb-like DNA-binding domain
	TraesCS5D02G326700	NB-ARC domain
	TraesCS5D02G326800	*Triticum monococcum* TmBAC 21C6 FR-Am2 locus, genomic sequence
	TraesCS5D02G326827	*Triticum aestivum* chromosome 3B-specific BAC library, contig ctg0954b
	TraesCS5D02G326900	*Triticum aestivum* cultivar Chinese Spring chloroplast, complete genome
AX-111118954		
	TraesCS5D02G327000	PREDICTED: *Aegilops tauschii* subsp. *tauschii* uncharacterized LOC109776055, mRNA
AX-108907798		

**Fig. 4:**
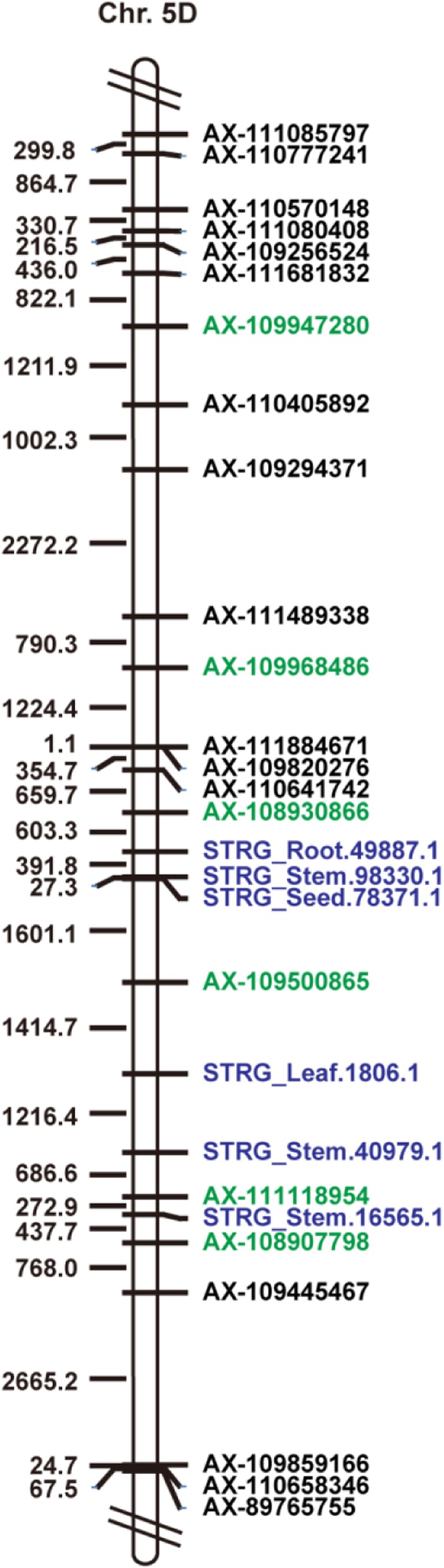
Diagram of a portion of chromosome 5DL. The long black rectangle represents the chromosome. The SNPs significantly associated with phenotype (green) and ncRNAs (blue) are indicated to the right of the chromosome. The physical intervals (kb) are shown on the left side of the chromosome

There were six lncRNAs located between SNPs AX-108930866 and AX-108907798 within a physical distance of 2.73 Mb on chr. 5DL, including STRG_Root.49887.1, STRG_Stem.98330.1, STRG_Seed.78371.1, STRG_Leaf.1806.1, STRG_Stem.40979.1, and STRG_Stem.16565.1 (Fig. 4). The sequences of these ncRNAs shared more than 90% identity (data not shown) with cytokinin oxidase/dehydrogenase genes (CKX2.3 and CKX2.4).

## Discussion

### The relationship between pleiotropy and phenotypic correlation

Three pleiotropic SNPs were identified in the study, which regulated two or three traits. Pleiotropy is a common phenomenon, and related traits are usually significantly correlated [22, 25]. For example, in barley, mutation alleles have pleiotropic effects on tillering and TGW traits [34]. In wheat, flag leaf length is correlated with yield-related traits [35]. It is well known that wheat yield is closely correlated with GNS, TGW, and number of effective spikes, and regions of chr. 5A and 6A were previously found to have pleiotropic effects on PH, grain yield, grain number, and TGW [28]. In our mutated population, GNS and NSL were correlated, which is consistent with previous results [36]. Previously, SNP markers *BS00022896_51* (located at 612 Mb on chr. 2A) and *wsnp_Ex_c40280_47375312* (located at 676 Mb on chr. 2A) were linked with GNS and NSL, respectively [36, 37], and in our study, another pleiotropic SNP, AX-109585477, was found to closly linked with these SNPs (695 Mb on chr. 2A) that also affects both GNS and NSL. Consistent with previous observations in rice [22], we found that PH was significantly correlated with FLA. Furthermore, SNP AX-109900989 was associated with both of these traits. MT and ET, which are two different indexes of tiller number, were also strongly correlated and shared the same candidate SNP, AX-89425861, on chr. 7D. These pleiotropic mutation alleles can be transferred into Kompetitive Allele Specific PCR (KASP) markers, which could be used to identify pyramided lines rapidly in earlier generations of segregating progenies, and will finally accelerate wheat breeding practice.

### Known and potentially novel mutation alleles associated with the investigated traits

The identified mutation alleles were classified into two types, one was known alleles, such as mutation alleles located at the ends of chr. 2BL and 5DL and on chr. 3BS and 3DL which reduced PH, and some of them were located close to those known regions. The dwarf gene *Rht4* was mapped to the terminus of 2BL, bin 2BL6–0.89-1.00 [38], which is from 444 to 739 Mb in the IWGSC Reference genome, and SNP AX-109900989 is located in this region at 679.5 Mb. SNP AX-111563435 is located close to a reported PH QTL on chr. 3B, QTL_height_3B_1 [39, 40]. QTLs for TGW were previously mapped to chr. 3BS, 4D, 5B, 5D, 6A, and 7B [20, 41–43], and two more mutation alleles affecting TGW were identified and they were mapped close to the known QTLs on chr. 3BS and 5DL [20, 42]. QTLs for tiller number were mapped to the terminus of 5AL [44], and a QTL on 5A affects tiller number [45]. The identified mutation allele on 5AL was also located at the 5AL terminus and might be the same as the tiller number QTL, but further phenotypic analysis of mutation lines and fine mapping data are needed to confirm this.

The other type was mutation alleles which did not colocalize with reported QTLs and might be novel alleles affecting PH-related traits. The mutation alleles on chr. 3DL have not yet been reported and might correspond to a novel gene. Genes and QTLs for tiller number have been mapped to chr. 1A, 1B, 1D, 2A, 2B, 2D, 3A, 4D, 5D, 6A, 6D, and 7A [43, 45–48], while our mutation alleles affecting MT and ET were located on chr. 4A, 6B, 7B, and 7D and did not colocalize with these previously mapped QTLs.

### Favorable mutation alleles and their potential application in future breeding programs

The candidate mutation alleles especially those of favorable alleles identified in the paper could enrich wheat genetic diversity. As different alleles and haplotypes of the target gene may have different effects on the phenotype [20, 49], breeders usually pyramid favorable alleles to improve target traits [30], such as plant architecture-related traits [33], so the identified favorable mutation alleles could be used in future wheat breeding programs. One potentially favorable allele is the G allele of AX-109900989, which reduced PH and FLA. Due to its effect on photosynthesis, changing FLA results in changes in yield [50]. For example, a leaf angle less than 25° enhances yield in wheat [51]. Thus, the G allele of AX-109900989 could potentially be used to decrease FLA and increase yield. Similarly, the T allele of AX-110371706, which increases SD, could also be used to increase yield. At the same time, unfavorable mutation alleles affecting TGW (G of AX-109326075 and T of AX-109947280) and tiller number (G of AX-89425861) should be avoided.

### The relationship between the terminal ends of chr. 3B and 5DL with plant height and thousand grain weight

Chromosome 3B is the largest chromosome in wheat, and multiple QTLs affecting plant architecture and yield-related traits have been identified in this chromosome [19, 39, 52] that are distributed on the entire chromosome. The 11 SNPs identified on chr. 3B (Fig. 1) were significantly associated with TGW, GNS, SD, PH, ET, or MT and, with the exception of SNP AX-109438215, which affects GNS, all colocalized with or mapped very near four previously reported meta-QTLs for yield-related traits. SNP AX-109326075 is located near the meta-QTL F6 (26.1–66.4 Mb) [53]. SNPs AX-109655447 to AX-111563435 colocalize with MQTL3B-3 (150.8–398.1 Mb), and SNPs AX-111029728 and AX-110536382 are both located near MQTL3B-4 (535.9–576.3 Mb) [39]. MQTL3B-3 and MQTL3B-4 are located in the same region as MQTL28 (116.8–737.0 Mb), which affects PH and yield-related traits [19].

Physical positions of both TGW and PH related SNPs identified on chr. 5DL in the study were colocalized with previously reported genes and QTLs. The TGW-associated SNP AX-109947280 identified in our study located at 409 Mb/566 Mb, while the cell wall invertase gene *TaCWI*, which is significantly associated with TGW, is located in this region [20]. This gene has been under strong selection during modern wheat breeding, and its genetic diversity has declined dramatically [20]. A QTL and a favorable allele affecting TGW are also located in this region (155.4 cM/170.7 cM and 421 Mb/566 Mb respectively) [41, 54]. Five SNPs associated with PH are located downstream of this region (414–419 Mb). The 42 genes located in this region included the Aux-IAA transcription factor, and a mutation in the auxin response gene resulted in decreased PH [55]; the PH of lines carrying these mutation SNPs were significantly lower compared with that of WT (Fig. 2a). Although the mutant SNPs did not occur in the genes, the five SNPs were very closely linked and the phenotypic variation might be caused by the genes in this region.

The six lncRNAs located near the four SNPs from AX-108930866 to AX-108907798 (about 6.6 Mb) (Fig. 4), are expressed in stems, leaves, or roots and share sequence similarity with CKX genes. CKX genes play important roles during plant development, and the level of CKX activity in barley, *Arabidopsis*, and tobacco results in variation in PH [56–59]. The dwarf gene *Rht23* is also located at the terminus of chr. 5DL [60]. So, it deduce that the terminus of chr. 5DL might play a critical role in wheat PH and TGW, but the relationship between our mutation alleles and *Rht23*, the CKX genes, and *TaCWI* needs to be further studied.

### Combining GWAS with *t*-tests could effectively reduce the false negative rate

Each advanced phenotypic mutants used in the study carried numerous mutations, it should think about how to rapidly identify linkage markers and mutation alleles affecting target traits. It is very difficult to use only a single mutant to identify linkage markers and mutation alleles affecting target traits, an alternative strategy is to use multiple mutants, as the probability of three individual mutants sharing a mutation in the same gene is less than 1E-05, and this probability is even lower when including more lines [61]. More than 67,000 qualified SNPs were used in the current study, and most of them should have no relationship with the target traits. It is very time consuming and unnecessary to analyze the association between each SNP and trait one by one, so TASSEL software was used to filter out most of the insignificant markers and to reserve as many potentially significant markers as possible by reducing the *P* value threshold from 7.4E-07 to 0.001. All potential markers were then further analyzed by *t*-tests to eliminate false positive markers.

Through the analysis of 10 plant architecture- and yield-related traits in 190 individual mutant lines, genome-wide association analysis and *t*-tests only gave similar results for PH, while for other traits, fewer than 40% of the markers were verified by *t*-test. This indicates that the set *P* threshold was appropriate, with fewer positive markers for PH or other traits omitted. This also means that if it only used genome-wide association analysis, the false negative rate would be very high, even with a relatively higher threshold. At the same time, the genetic effects analysis is based on the use of multiple mutant lines. Because mutation alleles present in only one or two mutant lines would be very difficult to identify, this would result in the identification of a certain number of false negatives. Some of the mutation alleles significantly associated with traits were identical with those identified in previous studies, and the favorable alleles could be used for wheat improvement.

## Conclusion

One hundred ninety advanced phenotypic wheat mutants were genotyped by 660 K SNP assay, and 10 agronomic traits were investigated under 5 environments. By using GWAS and *t*-tests methods, 32 SNPs distributed on 12 chromosomes were identified as mutation alleles associated with plant architecture and yield related traits, and chromosome 5DL clustered more alleles on PH and TGW. Among them, G allele of AX-109900989 could reduce PH and FLA, and T allele of AX-110371706 increased SD, those were favorable alleles. Five SNPs, AX-110409382, AX-109041501, AX-109446470, AX-111556361 and AX-89425861, were novel alleles associated with PH and tiller abilities. The mutants carrying favorable alleles could be further used in future breeding practice as diverse germplasm, and the SNPs could be converted into KASP markers and used to assist breeding selection.

## Methods

### Plant materials

Up to 20,000 seeds of an elite winter wheat (*Triticum aestivum* L.) cultivar Jing 411 were treated with 1.0 or 1.5% ethyl methanesulfonate solution according to the reported protocol [11] and 200 Gy or 250 Gy ɣ-rays every year. The germination percentage in the field ranged from about 60–70% by 1.0% EMS, 45–55% by 1.5% EMS, 50–60% by 200 Gy ɣ-rays, and 30–40% by 250 Gy ɣ-rays batch by batch. Each M_1_ plant was bagged at the heading and flowering stages to avoid hybridization and to strictly maintain self-crossing. The M_2_ populations were developed using both single-seed descent and mixed descent methods and each M_2_ population was kept at about 20,000 individual plants. Mutants affecting plant architecture- and yield-related traits were screened in the M_2_ generation in the field. Screening was continued in the next selfing generations until the phenotypes were stable. After a decade of continuous production in M_1_ and M_2_ populations, field selection, and identification, a mutant resource was constructed, which contained more than 4000 individual genotypes with phenotypic mutations. Within the mutant resource, a total of 190 advanced and stable independent individual mutant lines showing polymorphism in PH, tiller number, FLA, SL, NSL, SD, GNS, and/or TGW were selected for subsequent analysis, among them, 181 were induced by EMS and nine were induced by ɣ-rays.

### Phenotyping and data analysis

The mutants and the WT Jing 411 were planted at the headquarter (H) and Changping (C) experimental stations of the Institute of Crop Sciences, Chinese Academy of Agricultural Sciences in the 2014–2015, 2015–2016, and 2016–2017 growing seasons. Each genotype was planted in three rows with a row length of 2 m and an interplant distance of 5 cm. Two rows of plants that grew uniformly were selected for phenotyping. PH, tiller number, SL, and TGW were investigated in five different environments (2015H, 2015C, 2016H, 2016C, and 2017H); FLA, NSL, and SD were determined in four different environments (2015H, 2015C, 2016H and 2016C); and GNS was measured in two environments (2016H and 2016C).

Tiller number: PWT, MT, and ET were counted for all plants in each row at the end of November before the elongation stage and after the heading stage, respectively, and average PWT, MT, and ET per plant were calculated by dividing the total tiller number in a row by the seedling number of that row. The average PWT, MT, and ET values were used for subsequent analysis. Each genotype and environment had two replications. FLA between the flag leaf and its stem was measured 15 days after flowering using a protractor. Each genotype had 10 replications. NSL and GNS of the main spike for each genotype were counted in the field 20 days after flowering with five replications. PH, SL, and TGW were measured or calculated after harvest with five replications. SD was calculated by dividing NSL by SL.

Phenotypic data (Additional file 11: Table S8) were analyzed with QTL IciMapping v4.1 (http://www.isbreeding.net/) using the default settings to estimate the best linear unbiased estimate (BLUE) values for each environment and across environments. For calculating BLUE values, error variance of each environment is firstly calculated using model y = genotype+block+error, and then the phenotypic values of the individual in each environment are weighted and averaged by using the reciprocal of the error variance in each environment as the weight.

### Statistical analysis

Analysis of correlations between the investigated traits was performed using the BLUE values from QTL IciMapping v4.1. Calculation of standard deviations, *t*-tests, and average values of agronomic traits were performed using Microsoft Excel 2010.

### Genotyping

The 660 K SNP microarray was used to genotype the WT and 190 individual mutants. Microarray analysis was performed by China Golden Marker (Beijing) Biotech Co. Ltd. (CGMB, http://www.cgmb.com.cn), and the quality of the genotyping data was assessed. SNPs with a minor allele frequency (MAF) < 5% and a failed missing test (call rate < 97%) were excluded. A total of 67,402 SNPs were included in subsequent genome-wide association analysis.

### Linkage disequilibrium (LD) and population structure analysis

The LD across the chromosomes of the WT and 190 mutant lines was estimated using TASSEL 5.2 (https://tassel.bitbucket.io/) with 67,402 SNP markers (Additional file 12: Table S9). The squared allele frequency correlation (*r*^*2*^) was used for evaluation of LD [62] and the average *r*^*2*^ of 559,830 pairs of SNP markers at *P* ≤ 0.001 was calculated. PCA was conducted using TASSEL 5.2 for assessment of the population structure.

### Screening of candidate mutation SNPs

First, filtered SNPs and BLUE values for each trait were used in genome-wide association analysis performed using the mixed linear model (PCA + K) model of TASSEL v5.2 software [63] with the default settings. In order to reduce the risk of omitting positive candidate association markers, a less strict uniform threshold of *P* ≤ 0.001 was used to estimate additional potential significantly associated SNP markers. Manhattan plots were constructed using R 3.4.1 software (http://www.r-project.org/).

Secondly, mutant lines were classified into two groups based on the allele of a significant SNP identified by genome-wide association analysis, and *t*-tests were performed to test for significant differences (*P* ≤ 0.05) in the phenotypes of the two groups. When significant differences in phenotype were observed in more than 50% of environments, the SNPs were designated as candidate SNPs.

### Analysis of mutation allele effects

To identify target SNPs and mutation alleles leading to significant phenotypic variation, mutants carrying the mutant allele of a specific candidate SNP were identified, and variance between the phenotypes of WT and these mutants was further analyzed using *t*-tests.

### Sequence BLAST searches and gene annotation

The flanking sequences of the candidate alleles and reported QTLs, markers, and alleles were used as queries in BLAST searches against the IWGSC RefSeq V1.0 database (http://www.wheatgenome.org/) to determine their physical positions on chromosomes. Each candidate gene sequence was used as a query in a further BLAST search against the NCBI database (https://www.ncbi.nlm.nih.gov/) to annotate its function. Chromosome diagrams were drawn using mapdrawer [64].

## Supplementary information

**Additional file 1: Figure S1.** The principal component analysis with the variation partitioned between the first and the second principal components. The green circles indicate each subpopulation.

**Additional file 2: Table S1.** Significantly associated SNPs in each environment or across environments identified by genome wide association analysis.

**Additional file 3: Figure S2.** Manhattan plots showing the -log_10_(*p*) values from genome-wide association analysis of the investigated traits across environments.

**Additional file 4: Figure S3.** The QQ plots from genome-wide association analysis of the investigated traits across environments.

**Additional file 5: Table S2.** Plant height of mutant lines carrying mutation alleles of candidate SNPs.

**Additional file 6: Table S3.** Thousand grain weight of mutant lines carrying mutant alleles of candidate SNPs.

**Additional file 7: Table S4.** Maximum tiller number of mutant lines carrying mutant alleles of candidate SNPs.

**Additional file 8: Table S5.** Effective tiller number of mutant lines carrying mutant alleles of candidate SNPs.

**Additional file 9: Table S6.** Flag leaf angle of mutant lines carrying mutant alleles of candidate SNPs.

**Additional file 10: Table S7.** Spikelet density of mutant lines carrying mutant alleles of candidate SNPs.

**Additional file 11: Table S8.** Phenotypic raw data of PH, tiller number, SL, TGW, FLA, NSL, SD and GNS under each environment.

**Additional file 12: Table S9.** The alleles of WT and mutant lines with 67,402 SNP markers.

## Data Availability

All data generated or analyzed during this study are included in this published article and its supplementary information files.

## Abbreviations

BLUE: Best linear unbiased estimate
DArT: Diversity array technology
ET: Effective tiller numbers
FLA: Flag leaf angle
GNS: Grain number per spike
GWAS: Genome-wide association studies
KASP: Kompetitive allele specific PCR
LD: Linkage disequilibrium
MT: Maximum tiller numbers
NILs: Near isogenic lines
NSL: Spikelet number per spike
PCA: Principal component analysis
PH: Plant height
PVE: Percent variance explained
PWT: Pre-winter tiller numbers
Rht: Dwarfing genes
RILs: Recombinant inbred lines
SD: Spikelet density
SL: Spike length
SNP: Single nucleotide polymorphism
TGW: Thousand grain weight
TILLING: Targeting induced local lesions in genomes
WT: Wild type
