# Supplementary figures and images for: Functional mutation allele mining of plant architecture and yield-related agronomic traits and characterization of their effects in wheat
Source: BMC Genet. 2019 Dec 30;20:102. doi: 10.1186/s12863-019-0804-2 (PMC6937682; doi:10.1186/s12863-019-0804-2)

**Figure S1**

**
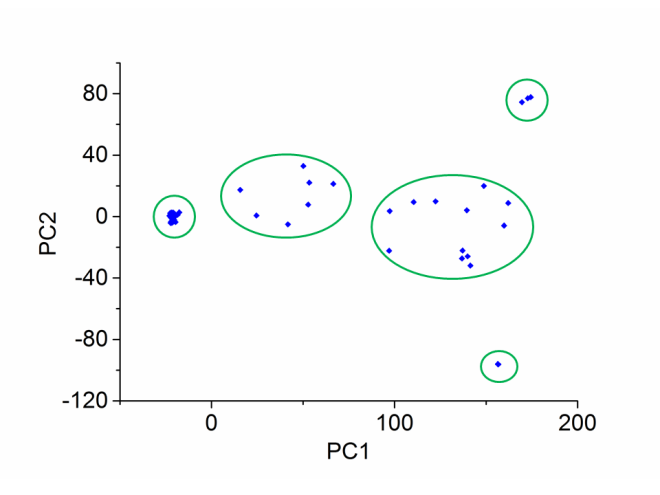
**

Supplement: Supplementary file 1 — Additional file 1: Figure S1. The principal component analysis with the variation partitioned between the first and the second principal components. The green circles indicate each subpopulation. [file 12863_2019_804_MOESM1_ESM.docx]

**Figure S2**


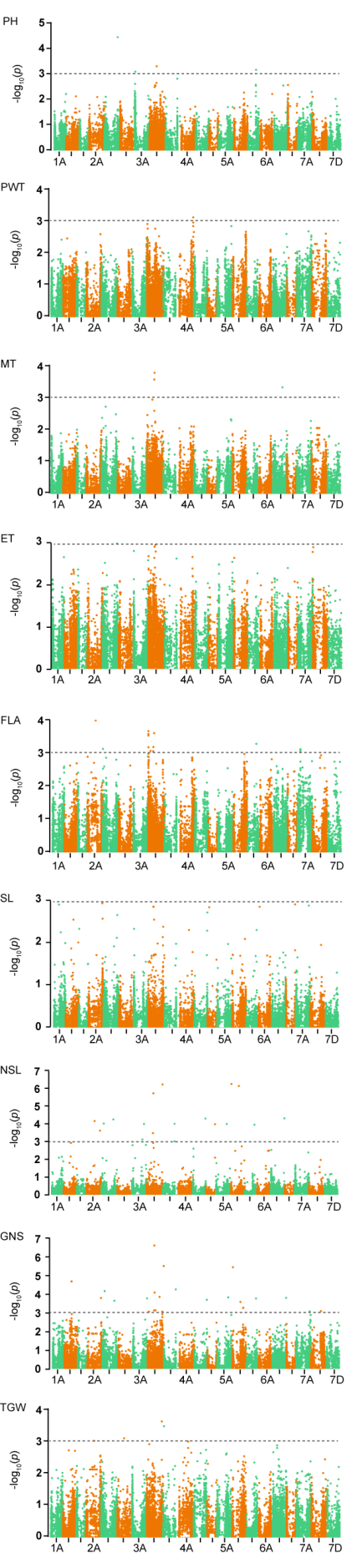

Supplement: Supplementary file 3 — Additional file 3: Figure S2. Manhattan plots showing the -log10(p) values from genome-wide association analysis of the investigated traits across environments. [file 12863_2019_804_MOESM3_ESM.docx]

**Figure S3**

**
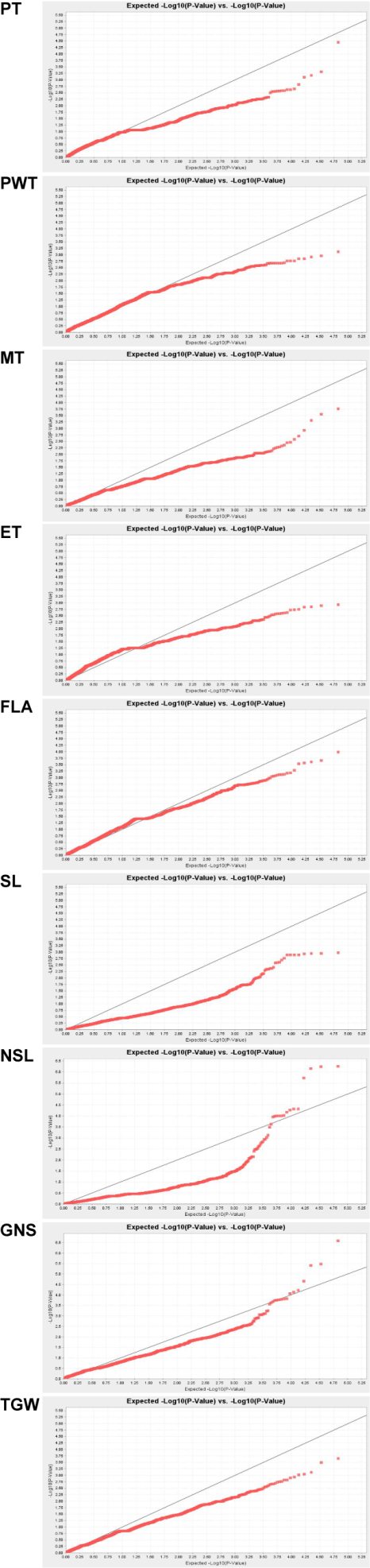
**

Supplement: Supplementary file 4 — Additional file 4: Figure S3. The QQ plots from genome-wide association analysis of the investigated traits across environments. [file 12863_2019_804_MOESM4_ESM.docx]
